# On-farm storage of livestock vaccines may be a risk to vaccine efficacy: a study of the performance of on-farm refrigerators to maintain the correct storage temperature

**DOI:** 10.1186/s12917-018-1450-z

**Published:** 2018-04-19

**Authors:** Paul D. Williams, Gustavo Paixão

**Affiliations:** 1 MSD Animal Health, Walton Manor, Walton, Milton Keynes MK7 7AJ UK; 2 School of Veterinary Science, University of Bristol, Langford House, Langford, Bristol BS40 5DU UK; 3 Present address: Animal and Veterinary Research Centre (CECAV), Universidade de Trás-os-Montes e Alto Douro, 5001-801 Vila Real, Portugal

**Keywords:** Vaccine storage, Cold chain, Veterinary vaccines, Livestock, Temperature monitoring

## Abstract

**Background:**

Livestock vaccines (LV) are often stored on-farm, in a refrigerator (fridge), prior to use and little is documented about the storage conditions during this period. As the quality of a vaccine can be impaired by storage at an incorrect temperature, the present study aimed to evaluate the on-farm performance of farm fridges to maintain the correct storage temperature. From January to August 2014, temperature data loggers were placed on selected farm fridges used to store LV (*n* = 20) in South-West England.

**Results:**

Temperature recording data was available from 17 of the 20 farms. Fifty-nine percent of farm fridges had at least one temperature recording above 8 °C, 53% had at least one recording below 2 °C and 41% at or below 0 °C. Internal fridge temperatures attained 24 °C and dropped to − 12 °C as an absolute maximum and minimum respectively. Fridges tested spent an average of 16% of the total time recorded above 8 °C. Time of the year significantly influenced the percentage of time above 8 °C. External and internal temperatures were found to be positively correlated (*p* < 0.001). Statistical significant differences in internal and external temperatures were found between March and August.

**Conclusions:**

The majority of fridges in this study would have failed to keep any stored LV within the recommended storage temperature range. If LV are going to be stored on-farm prior to use, then urgent improvements in this part of the cold-chain are required in order to insure vaccine efficacy is not compromised.

## Background

Livestock vaccines (LV) are crucial tools for animal and public health. They are a cost-effective method to prevent animal disease, enhance the efficiency of food production, and reduce or prevent transmission of zoonotic and foodborne infections to people [1]. Proper usage and storage of LV are essential to achieve maximal vaccine safety and efficacy [2]. Vaccines should always be stored as per manufacturer instructions and general recommendations [3]. For the majority of vaccines, especially those used in cattle, sheep and pigs, the recommended storage temperature (RST) is between 2 °C and 8 °C [4]. Vaccines stored at different temperatures could result in potency loss and impaired efficacy [5–7]. Furthermore, emphasis is being increased nowadays on vaccine management to protect vaccines from freezing [8, 9] as cold chain practices tend to prioritize protecting vaccine from heat damage. Very often the risk of vaccine exposure to temperatures below freezing point is considered greater than heat exposure [10–12].

Vaccines thermal sensitivity can be explained by the complex nature of the three-dimensional structure of the antigen [13]. There are clear data regarding effects of temperature oscillation in human vaccines and therefore multiple recommendations to ensure optimal potency [14]; but there is little information regarding the tolerance levels for most commonly used LV, except for some specific vaccines, like the foot and mouth disease [7]. Vaccines can be classified into two general groups: inactivated and live vaccines. Live vaccines are made of weakened, attenuated versions of infectious viruses, bacteria, fungus or parasites that can replicate in vivo. Live vaccines do not normally require adjuvants to boost the immune response but are more sensitive to potency loss during storage and distribution, especially at elevated temperatures [13]. Inactivated vaccines on the other hand often require adjuvants to boost the immune response. They are typically more stable to moderate heat exposure than live vaccines, yet more sensitive to freezing [15], especially when containing adjuvant with aluminum salts, which may aggregate on freezing, lowering the adjuvant effect [16, 17].

The end of the cold chain, where the vaccine is stored prior to use, is pointed as a critical stage due to the widespread use of domestic refrigerators, especially in developing countries [18]. To address this issue, World Health Organization (WHO) establishes guidelines for manufacturers of refrigerators intended to store vaccines to use in humans in order to ensure performance standards [19]. Stand-alone units, freeze or refrigerate only, and frost-free or automatic defrost cycle are important features on units intended to store vaccines. Ideally, units should be dedicated to storage of vaccines and temperature monitoring should be performed with calibrated devices and digital data loggers [20]. In regards to animal health, there are no guidelines available in terms of refrigerators requirements.

In the United Kingdom, LV are often stored on-farm, in domestic refrigerators, prior to use and administered by farm workers [21, 22]. The objective of the present study is to evaluate the LV storage associated risk when stored on-farm before use.

## Methods

### Data recording

Between 15th January and 25th August 2014, 3 temperature data loggers (EL-USB-2-LCD, Lascar Electronics, UK) were each placed on 20 non-randomly selected farms from 3 large animal veterinary practices in South West England (Somerset, Devon and Dorset). Two data loggers were placed inside of the fridge used to store LV used on the farm. One data logger was placed on the uppermost shelf of the fridge, the other on the lowest. Both data loggers were placed towards the back of the fridge. The remaining data logger was placed outside of the fridge, but in the same room as where the fridge was located. The data loggers were setup to record the temperature every 30 min after placement. At each farm the approximate number of animals on the farm and an estimated age of the fridge was recorded, and if the fridge was used for any other storage purposes beside that of LV.

### Data analysis

Descriptive statistical analysis was performed in Microsoft Excel 2010 Microsoft Corporation, Redmond, WA, USA, 2010. One-way variance and regression analysis was done in JMP, Version 7.0. SAS Institute Inc., Cary, NC, 2007. Pearson’s correlation factor Tukey’s test was used to compare means when significantly different (*p* < 0.05).

Further analysis of the individual data logger temperature recordings from inside the fridges was undertaken using a script written using Microsoft Visual Basic for Applications, Microsoft Corporation, Redmond, WA, USA, 2010. This script processed all individual temperature recordings for each internal data logger and categorised them into “Events”. An Event occurred when two or more consecutive temperature recordings were below or above a defined temperature parameter: above 8 °C, below 2 °C and equal or below 0 °C; meaning there could be up to 3 different Event types for each data logger; “Above 8 °C”, “Below 2 °C” and “At or below 0 °C”. The number of consecutive 30-min recordings outside the temperature parameter determined the Event duration. Each Event occurrence would provide an opportunity for any vaccine stored in the fridge at that time to equilibrate with the temperature in the fridge.

## Results and discussion

Of the 20 farms, complete temperature recordings were available from 17 farms. On Farm 1 the data loggers from inside the fridge were missing at the end of the study, Farm 8 stopped storing vaccines on-farm and Farm 15 could not be contacted after initially agreeing to take part in the study. Dairy farms composed the majority of farms present in the study (8/17) (Table 1). Of those, 75% of fridges were located in the office adjacent to the milking parlour. All fridges present in the study were classified as common domestic fridges, with no power backup or temperature logger. Dairy farms also had a higher proportion of older fridges (5/8) compared to other farm types (3/9). Seventy-one percent (12/17) of the fridges tested were not dedicated to vaccine storage. This was most noticeable in the beef and sheep farms, where the fridge was also used for domestic purposes. When possible, farmers should have a dedicated vaccine storage refrigerator. This could minimize the frequency of openings, reducing temperature fluctuations, and ensure appropriate biosecurity [20].

**Table 1:** Farm and fridge details for which temperature recordings were available (*n* = 17)

Farm No	Farm details		Fridge details			
	Type	No Animals	Age (Years)	Location	Vaccine only	Other use
2	Dairy	140 cows	< 10	Farm office	Yes	–
3	Dairy	250 cows	> 10	Farm washroom	No	Colostrum, milk test kits
4	Dairy	280 cows	< 10	Parlour office	Yes	–
5	Dairy	350 cows	< 1	Parlour office	Yes	–
6	Dairy	420 cows	> 10	Parlour office	Yes	–
7	Dairy	270 cows	> 10	Parlour office	No	Medicines
9	Dairy	500 cows	> 10	Parlour office	No	Medicines
10	Dairy	250 cows	> 10	Parlour office	No	Drinking water
11	Pig	2000 sows	> 10	Workshop	No	Medicines
12	Pig	950 sows	< 10	Staff room	Yes	–
13	Sheep	500 ewes	< 10	House kitchen	No	House food
14	Beef and Sheep	360 cows &1700 ewes	> 10	House porch	No	Beer
16	Beef and Sheep	50 cows & 500 ewes	< 10	House outside larder	No	Household food
17	Sheep	370 ewes	< 5	House kitchen	No	Household food
18	Sheep	100 ewes	< 5	House kitchen	No	Household food
19	Sheep	60 ewes	> 10	House kitchen	No	Household food
20	Beef and Sheep	60 cows & 320 ewes	< 10	House kitchen	No	Household food

**Fig. 1:**
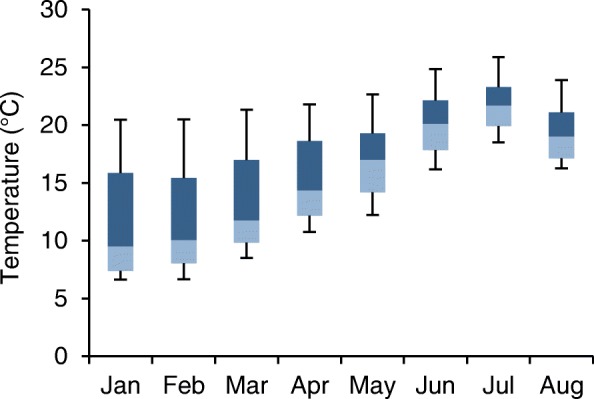
Box plot showing farm variation in mean monthly external temperature. Boxes represent interquartile range and whiskers span all data points within 1.5 times interquartile range of the nearer quartile

The minimum and maximum external temperatures recorded during the study period were 1.5 °C in January and 33.0 °C in July respectively. There was a marked variation in the mean external temperatures recorded between each farm, on a monthly basis; as would be expected, there was an increase in the median external temperature as the study progressed from January to August (Fig. 1). All fridges were located in the same geographical region so the marked variation in monthly temperatures was mostly likely due to their individual location on farm. A fridge is designed to operate within a specific temperature range and if placed in an environment where the external temperature is outside this range it may not operate as intended. A properly functioning refrigerator can only lower the temperature of its internal environment, not raise it. As the external temperature during the study did not drop to 0 °C or below, it was not possible to observe what impact such a low external temperature might have had on the internal temperature of a fridge.

**Fig. 2:**
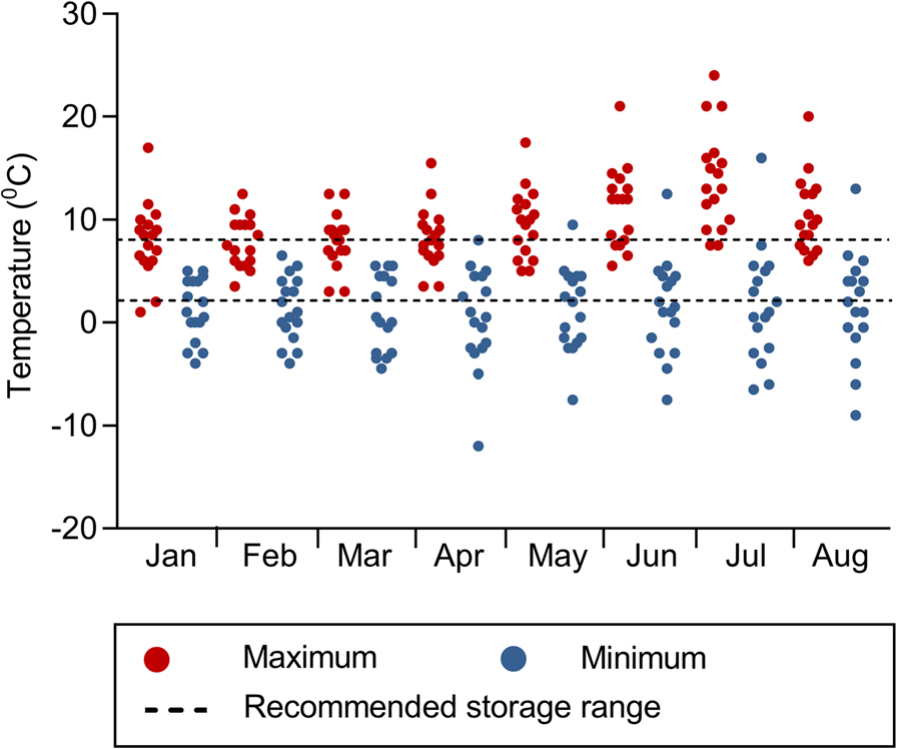
Maximum and minimum internal fridge individual temperatures recorded on a monthly basis

Figure 2 shows the maximum and minimum internal fridge temperatures recorded. Throughout every month of the study, an average of 59% (10/17) of the farms had at least one temperature recording above 8 °C, 53% (9/17) had at least one recording below 2 °C and 41% (7/17) at or below 0 °C. The maximum internal fridge temperature appeared to be affected by the external temperature, with an increase in the number of fridges recording at least one internal temperature greater than 8 °C between May and August. Within those recordings, temperatures had reached 24 °C, as a maximum recorded temperature in July on Farm 9, and dropped to − 12 °C, as minimum recorded temperature in April, on Farm 16. Even though some of these individual recordings do not correspond to sustained events, it is clear that temperature can deviate considerably from the RST.

**Fig. 3:**
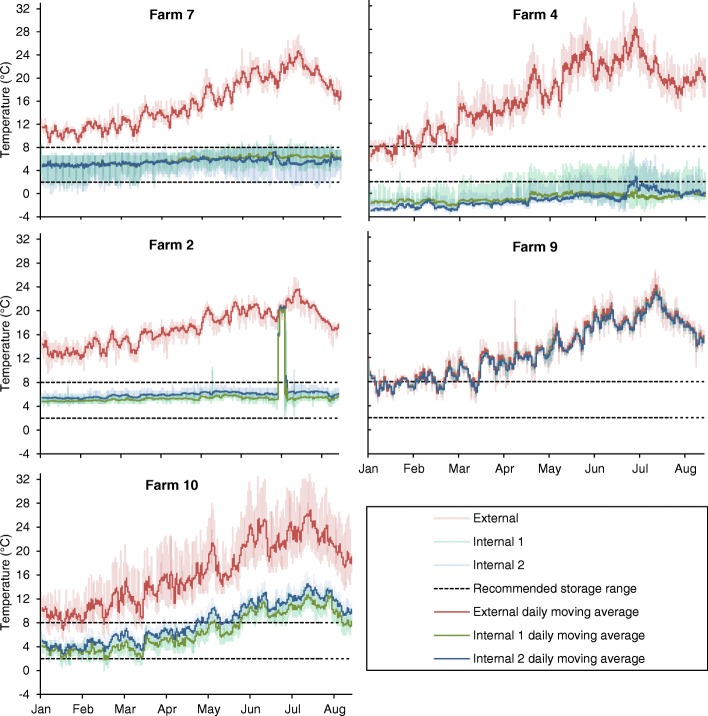
Temperature recordings from five selected farms. Shaded areas represent individual recordings whereas the solid-line represents a daily moving average. External is the data logger placed outside of the fridge, Internal 1 the data logger placed on the uppermost shelf in the fridge and Internal 2 the lowermost. Squared dot line represents the recommended storage temperature range of 2 to 8 °C

Figure 3 displays a graphical presentation of the temperature recordings from a selection of farms. Farm 7 had one of the better performing fridges during the study period. Although there are individual temperature recordings outside the 2 and 8 °C storage range, the trend in temperature recordings is between these two values, as demonstrated by the daily rolling average. The Farm 2 fridge also performed well up to the point of a dramatic temporary increase in internal temperature recordings from 04:45 on 11th July to 00:45 on 16th July. Retrospective questioning of farm staff did not identify the cause of this, but either the fridge door being left open or an interruption to the fridge power supply seem most likely. The Farm 10 fridge internal temperature increased in line with increasing external temperature. As already noted, this increase in fridge temperature with increasing external temperature was recorded in several other fridges. The Farm 4 fridge recordings were consistently well below 0 °C for much of the study period. The Farm 9 fridge internal temperature tracked the external temperature throughout the study period. The farm staff were notified that the fridge did not appear to be working, despite being plugged in, at the start of the study period but it was not replaced during the study. In different degrees, external temperature seems to have affected the internal temperature of the fridges tested.

Fridges subject to study recorded sustained temperatures above and below the RST (Table 2). Fridges tested on our study spent an average of 16% of the total time recorded above 8 °C. Time of the year significantly influenced the percentage of time above 8 °C (*p* = 0.0135, *n* = 136); summer months contributed more for this phenomenon: during July and August, the farm fridges had spent an average of 31% of their time above the RST, contrasting with 5% during January and February. Moreover, the same units tested spent an average of 16% of the time below 2 °C and 9% at or below 0 °C. These results suggest that external temperatures are positively correlated with the percentage of time recorded above 8 °C (*r* = 0.2523, *p* = 0.003, *n* = 136) and negatively correlated with the percentage of time below 2 °C (*r* = − 0.2271, *p* = 0,008, *n* = 136). Table 3 shows significant differences (*p* < 0.05) in external and internal temperatures between months, except for January and February (*p* = 0.2027), where external and internal temperature means were similar. Although significant differences in monthly external temperatures are expected, following environmental temperature fluctuations in temperate climates, internal fridge temperatures shouldn´t have been significantly different. This finding can be explained by the inability of the fridges to maintain their internal temperature within the RST. Furthermore, monthly internal temperature means tend to follow external temperatures means. Contrarily, internal temperature SD appear to be negatively correlated with external temperatures SD (*r* = − 0.8752, *p* = 0.0044, *n* = 8). This may be due to the higher variance of a fridge’s capability to maintain internal temperature within the RST on hotter months. The higher external temperature SD observed on cooler months could be explained by the higher temperature differences recorded amongst the rooms where the fridges were located.

**Table 2:** Summation of all Events, expressed as percentage (%) of the total minutes recorded in a given month. Data is only shown for farms for which there was at least one Event occurrence

Farm No	Jan	Feb	Mar	Apr	May	Jun	Jul	Aug
a) Events above 8 °C								
2							17%	
6		1%			2%	13%	53%	69%
9	26%	46%	59%	99%	100%	100%	100%	100%
10					45%	97%	100%	97%
11	29%	16%	5%		2%			
12		1%				2%	27%	39%
13				9%	20%	34%	31%	68%
16			14%				10%	48%
18	4%	4%	14%	16%	43%	69%	55%	37%
19	33%	19%	6%	24%	85%	100%	100%	99%
b) Events at or below 0 °C								
3	54%	38%	16%	7%	18%	14%	1%	
4	98%	97%	95%	94%	87%	81%	80%	87%
11					1%	1%	1%	1%
13							5%	1%
14	100%	87%	60%	15%	6%			
16				4%	25%	54%	41%	14%
20			1%	1%			1%

**Table 3:** Descriptive statistics of external and internal temperatures within the different months. Means with different superscript letters in a column are significantly different (*p* < 0.05)

		External temperature			Internal temperature		
Month	*n*	Mean	SD	SEM	Mean	SD	SEM
Jan	13,447	11.8^a^	5.1	0.044	4.1ª	2.8	0.024
Feb	24,192	11.9^a^	5.0	0.032	4.0^a^	2.8	0.018
Mar	26,784	13.4^b^	4.8	0.030	4.2^b^	2.9	0.018
Apr	25,920	15.2^c^	4.0	0.025	4.6^c^	3.0	0.018
May	26,784	17.0^d^	3.8	0.023	5.0^d^	3.2	0.012
Jun	25,920	20.3^e^	3.4	0.021	5.7^e^	4.0	0.025
Jul	26,784	21.8^f^	3.2	0.019	6.2^f^	4.7	0.028
Aug	26,265	19.1^g^	3.1	0.019	5.9^g^	3.9	0.024
*p* value		< 0.05			< 0.05		

The fact that farms were not randomly selected predisposes the study to selection bias. Also, as the study was undertaken for only part of the year, it was not possible to determine how the remaining months would have impacted the results. The number of farms that participated was limited, making the study subject to minor statistical confidence. However, the group of farms included in the study was broad spectrum in the sense they include different farmed species and stock sizes, reflecting the reality of South West England. Finally, this study only shows temperature variances when stored in a farm fridge and vaccines can be exposed to sub-optimal temperatures during transportation and administration. The period of 30 min between recordings was chosen as the most frequent interval available to capture temperature recordings throughout the study period, based on data logger storage capacity. Personal communication with the Technical Department, Lascar Electronics, indicated it typically takes 20 min for the contents of a normally packaged vaccine vial to equilibrate with its external environmental temperature. Based on this, a minimum possible duration of 30 min, was established to categorize Events. Each Event occurrence would provide an opportunity for any vaccine stored in the fridge at that time to equilibrate with the temperature in the fridge.

Despite the limitations, the findings from this research confirm anecdotal reports that on-farm vaccine storage is often being neglected by UK farmers. Improvements related with on-farm refrigerators used to store vaccines are strongly advised. Many of these improvements can be easily done without great financial investment, but educating and motivating farmers for the need to change is required. Minimizing the vaccine storage time on-farm should be promoted by veterinarians and suitably qualified persons.^1^ In situations where vaccine is being stored on farm then the routine temperature monitoring of fridges is recommended. This could be done with simple maximum-minimum thermometers or digital loggers similar to those used in this study, but both must be checked on at least a daily basis. Digital loggers are available with buffered probes, which can send automatic alerts remotely via SMS or email to forewarn of potential temperature deviation, therefore, allowing action to be taken before a vaccine is exposed to a potentially damaging temperature. Although these are more expensive, their use could be justified where larger quantities of vaccine are being stored. Vaccine vial monitors, visual freeze indicators or a “shake test” may also be used to determine if a vaccine has been exposed to potentially damaging storage temperatures. Vaccine vial monitors trace if the vaccine has suffered cumulative heat impact by showing clear visual change; the reactions vary in accordance with the category of vaccine to which they are assigned [14]. Visual freeze indicators provide a visual indication of exposure to freezing [23]. Additionally, the “shake test” enables visual detection of whether an aluminium-based freeze-sensitive vaccine has been affected by freezing [24].

In this study, it was also demonstrated that external temperatures influence a fridge’s internal temperature and consequently the ability of the fridge to maintain the temperature within the RST. Therefore, placing the farm’s fridge in a controlled temperature room could improve a fridge’s capability of stabilizing its internal temperature. The results from this research go in line with other recent studies where weaknesses of domestic fridges used to store human vaccines, a common finding in developing countries, were also been found [9, 25]. More comprehensive studies, with a larger number of fridges are needed in order to fully understand the scale of the problem.

## Conclusions

All of the fridges in this study failed to maintain their internal temperature within the RST, at some point during study period. The internal temperature recorded and the total time recorded outside the RST were influenced by external temperature. More importantly, the majority of fridges present in this study were inadequate to provide proper vaccine storage, having had at least one sustained event outside the RST. In sum, LV are at risk of losing potency and efficacy when stored on-farm prior to use. Despite the many variables that play a crucial role in farm animal vaccination, there is a real need to improve on-farm vaccine storage conditions or implement alternative measures in order to minimize the risk of a vaccine losing potency and to ensure its retains efficacy.

## Abbreviations

LV: Livestock vaccines
RST: Recommended storage temperature

## Footnotes

1 Suitably qualified person (SQP) is a phrase used to describe a person, without a degree in veterinary or pharmacy, who is permitted to prescribe and supply veterinary medicines classified as prescription-only (POM-VPS and NFA-VPS), in the UK.
